# Predicting Potential Habitats of the Endangered Mangrove Species *Acanthus ebracteatus* Under Current and Future Climatic Scenarios Based on MaxEnt and OPGD Models

**DOI:** 10.3390/plants14182827

**Published:** 2025-09-10

**Authors:** Jiaqi Chen, Liuping Wu, Chongcheng Yang, Qiongzhen Qiu, Yi Wang, Zhixin Li, Chunhua Xia

**Affiliations:** College of Coastal Agricultural Sciences, Guangdong Ocean University, Zhanjiang 524088, China; 15989097775@stu.gdou.edu.cn (J.C.); wuliuping@gdou.edu.cn (L.W.); 15014960216@stu.gdou.edu.cn (C.Y.); 18718656183@stu.gdou.edu.cn (Q.Q.); wang1206282637@outlook.com (Y.W.); 13600438967@163.com (Z.L.)

**Keywords:** climate change, *Acanthus ebracteatus*, MaxEnt model, OPGD model, suitable habitat

## Abstract

Climate change threatens coastal biodiversity, necessitating proactive conservation for endangered species like the mangrove *Acanthus ebracteatus.* This study integrated the MaxEnt and OPGD models to simulate its potential suitable habitats under current and three future SSP scenarios (SSP126, SSP245, and SSP585). Based on the MaxEnt model, sea surface salinity (SSS_range), sea surface temperature (SST_max), soil texture (T_silt, T_sand), and annual precipitation (Bio12) were identified as the dominant factors influencing its distribution, with SSS_range emerging as the key constraint. Furthermore, interaction analysis using the OPGD model revealed significant synergistic effects, particularly between salinity and soil properties (q > 0.8), underscoring the importance of multi-factor interactions in ecological niche modeling. Under the three SSP scenarios, the suitable habitat is projected to expand northeastward, accompanied by a poleward shift in the distribution centroid, driven predominantly by warming temperatures and altered rainfall patterns. KDE analysis revealed that existing protected areas do not fully cover regions with high habitat suitability. We propose a stratified conservation strategy that enhances in situ protection in core zones, initiates assisted restoration in potential habitats, and promotes experimental outplanting in future climatically suitable areas. This study provides scientific insights for the conservation and management of *Acanthus ebracteatus* under global climate change.

## 1. Introduction

With the increasing frequency of extreme weather events and the escalating threat of sea-level rise, research focused on conserving coastal protective vegetation (particularly mangroves) and predicting potential habitats has become critically important [1]. As the dominant coastal forests in tropical and subtropical regions, mangroves provide critical short-term protection against typhoons and tsunamis, while also offering essential long-term ecological value through enhanced resilience to sea-level rise [2]. However, mangrove ecosystems globally face extinction threats driven by anthropogenic development, climate change, extreme natural events, and cumulative ecological degradation [3]. Among the 70 species of true mangrove plants worldwide, 11 species (16%) of mangrove plants are listed as Critically Endangered (CR), Endangered (EN), or Vulnerable (VU) [4]. In China, habitat degradation has resulted in approximately 50% of both native true mangrove and semi-mangrove species being classified as rare and threatened to varying degrees. *Excoecaria agallocha* and *Acrostichum aureum* have become extinct in Fujian Province, *Rhizophora stylosa* in Hong Kong, and *Ceriops tagal* in Guangxi. Furthermore, naturally occurring populations of *Rhizophora stylosa*, *Sonneratia × hainanensis*, and *Sonneratia ovata* in Hainan Province are now on the verge of extinction [5].

*Acanthus ebracteatus* Vahl. is a true mangrove species of the genus *Acanthus* (Acanthaceae), which is an evergreen subshrub that is naturally distributed in the high-tide zone areas of the southeast coast of India and the northwest coast of Oceania [6]. *A. ebracteatus* generally grows on the inner edge of mangrove forests or on the sides of tidal channels, sometimes forming small areas of pure forests stands. Its scrub structure provide unique microhabitats for crabs and small fishes, and its flowers serve as nectar sources for coastal pollinators, occupying a special ecological niche and holding significant ecological value [7]. *A. ebracteatus* is also widely recognized for its unique biological activities, including antithrombotic, anti-aging, antagonistic to phytopathogenic bacteria, and antitumor properties [8]. Due to global climate change and human activities, *A. ebracteatus* is classified as Endangered (EN) in China according to the IUCN criteria [9]. To protect this cherished and endangered mangrove species, researchers have investigated the current status of its resources in Guangxi and the Leizhou Peninsula and have developed key propagation techniques to aid in its conservation [10,11,12]. However, the prediction of a potential suitable habitat in existing studies remains vague, and the identification of its key environmental factors lacks in-depth exploration.

Ecological Niche Modeling (ENM) is a method commonly used in ecology and conservation biology to predict species’ potential distribution and habitat suitability by integrating known species occurrence records with environmental variables [13,14]. Among ENM techniques, the Maximum Entropy (MaxEnt) model [15] is widely employed in studies of endangered species [16,17] due to its reliable predictive performance even with small or irregularly sampled datasets [18]. In recent years, the MaxEnt model has also been applied in studies supporting mangrove restoration [19,20,21]. However, most of these studies have focused on widespread species or mangrove communities as a whole, leaving a considerable gap in the systematic understanding of the distribution patterns and climate change responses of rare species such as *A. ebracteatus*. Moreover, conventional ENM studies often focus on the individual effects of environmental variables, overlooking the interaction effects among them. In mangrove ecosystems, which are shaped by complex interactions among factors such as salinity, temperature, and precipitation, neglecting these interactions may lead to an oversimplified understanding of actual habitat requirements.

To address these limitations, this study integrate the MaxEnt model with the Optimal Parameter-based Geographical Detector (OPGD) model [22]. MaxEnt predicts a potential suitable habitat for *A. ebracteatus* in China, while Kernel Density Estimation (KDE) is used to identify distribution hotspots and conservation gaps [23]. The OPGD model then pinpoints the key environmental drivers and quantitatively assesses their interaction effects. This combined methodology helps not only in delineating potential habitats but also deepens the mechanistic understanding of why certain areas are suitable by revealing interactions among environmental factors. Through these analyses, we aim to develop a more comprehensive understanding of the habitat requirements of *A. ebracteatus*, simulate dynamics of suitable habitats under future climate scenarios, and identify conservation-priority areas. The results are expected to provide a more robust scientific basis for the conservation, management, and targeted restoration of the endangered mangrove species *A. ebracteatus* in China.

## 2. Results and Analysis

### 2.1. Model Optimization and Accuracy Evaluation

The default parameter settings of the MaxEnt model are a regularization multiplier (RM) of 1 and feature classes (FC) of LQHPT. To enhance model robustness and mitigate overfitting, we optimized the MaxEnt parameters beyond the default settings using the ENMeval package [24]. The optimal parameter combination was selected based on the minimum delta.AICc value. The results show that delta.AICc = 0 when RM = 2 and FC = LQ (Supplementary Figure S1a), which indicates the most parsimonious model, reducing the difference between training and testing AUC (AUC.diff) by 13.81% and effectively mitigating overfitting (Supplementary Figure S1b).

The optimized MaxEnt model demonstrated a high predictive accuracy. Across 10 replicate runs, the mean AUC for the training set was 0.883 ± 0.034, while for the test set, it was 0.858 ± 0.053. Both values are well above 0.85 and significantly outperform a random prediction (AUC = 0.5), confirming the model’s robust predictive ability for *A. ebracteatus*.

### 2.2. Key Environmental Factors That Are Drivers of Habitat Distribution

#### 2.2.1. Identification and Ranking of Dominant Factors

We identified the dominant environmental factors driving the potential habitat distribution of *A. ebracteatus* by assessing their percent contribution and permutation importance (Table 1). Five key variables emerged as the most influential determinants: Range of Sea Surface Salinity (SSS_range), maximum sea surface temperature (SST_max), Topsoil Silt (T_Silt), Topsoil Sand (T_Sand), and annual precipitation (Bio12). Together, these factors accounted for 90.9% of the total percent contribution and 80.4% of the permutation importance, underscoring their dominant role in shaping distribution patterns. Among these, SSS_range was the primary limiting factor, contributing 36.4% individually and ranking highest in permutation importance, followed by T_Silt and SST_max.

The Jackknife test provided further validation of these variables’ roles. When variables were used in isolation, SSS_range, T_Sand, SST_max, and T_Silt yielded the highest model gains (Supplementary Figure S2a). This test also showed that T_Sand, SSS_range, Elevation (Elev), Bio12, and SST_max achieved the highest AUC values, confirming their significant individual predictive power (Supplementary Figure S2b). Crucially, it was revealed that excluding SSS_range caused the sharpest decline in regularized training gain, followed by T_Silt, confirming their substantial explanatory power for predicting distribution.

#### 2.2.2. Ecological Response Mechanisms of Dominant Factors

To elucidate the ecological mechanisms underlying habitat suitability, we analyzed the response curves of the dominant environmental factors, considering a habitat probability above 0.5 as the threshold for a suitable habitat (Supplementary Figure S2c–g). Habitat suitability for *A. ebracteatus* was constrained by distinct thresholds and optimal ranges across marine, soil, and climatic variables. Among the marine factors, suitability exhibited a strong negative relationship with the SSS_range, remaining favorable below 4.6 PSU (Supplementary Figure S2c). In contrast, suitability was positively correlated with SST_max, reaching an optimum within the 30.92–32 °C thermal range (Supplementary Figure S2d). For soil properties, the response to T_Silt was unimodal, with suitability peaking at a probability of 0.78 when silt content was 46.4%, reporting an optimal range of 30.9–50.5% (Supplementary Figure S2e). Conversely, suitability declined sharply as T_Sand content increased, falling below the 0.5 threshold when it surpassed 41.1% (Supplementary Figure S2f). Lastly, suitability showed a positive association with Bio12, with an optimal range of 1750.2–2901.8 mm (Supplementary Figure S2g). Collectively, these findings demonstrate that the potential distribution of *A. ebracteatus* is co-shaped by the interplay of marine and terrestrial drivers, underscoring salinity and soil texture as the primary limiting factors defining its ecological niche.

#### 2.2.3. Interaction Effects of Environmental Factors Revealed by OPGD Analysis

To better understand the driving mechanisms behind the spatial patterns of suitable habitats for *A. ebracteatus*, we applied the Optimal Parameter-based Geographical Detector (OPGD) model. This method provides a quantitative framework to assess not only the individual contribution of each environmental factor but, more importantly, to identify and measure the interactive effects between factors that collectively influence species distribution.

**Figure 1:**
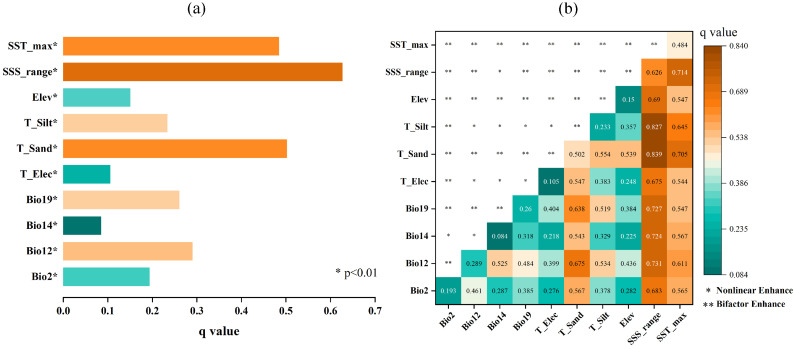
OPGD analysis of the spatial differentiation of *Acanthus ebracteatus* potential habitats. (**a**) Factor detector: the individual explanatory power (q-value) of each variable. (**b**) Interaction detector: the type and strength of interactive effects between factor pairs.

The factor detector analysis confirmed that all environmental variables had statistically significant effects on habitat spatial differentiation (*p* < 0.01). SSS_range showed the strongest individual explanatory power (q-value 0.63), followed by T_Sand and SST_max, reinforcing the results obtained from the MaxEnt model (Figure 1a).

More significantly, the interaction detector analysis revealed that the combined effect of any two environmental factors significantly exceeded their individual effects. The dominant interaction type was bifactor enhancement [q (X_1_ ∩ X_2_) > Max (q (X_1_), q (X_2_))], indicating synergistic effects where factors work together to amplify their impact on habitat distribution. Particularly, interactions involving SSS_range consistently yielded the highest q-values. Its interaction with soil factors, in particular, showed a significant synergistic enhancement effect (q > 0.8), emphasizing its central role in interacting with other variables to drive spatial patterns of habitat suitability (Figure 1b).

These OPGD results provide important insights into the complex ecological mechanisms governing the distribution of *A. ebracteatus*. They demonstrate that the species’ habitat suitability is determined not simply by the additive effects of individual factors but through complex nonlinear interactions between variables, with salinity variation serving as a key modulator in these interactive effects. This comprehensive analysis addresses the potential limitations of using MaxEnt alone by revealing the interdependent nature of environmental drivers that collectively determine species distribution patterns.

### 2.3. Current Distribution Pattern of Potentially Suitable Habitats

**Figure 2:**
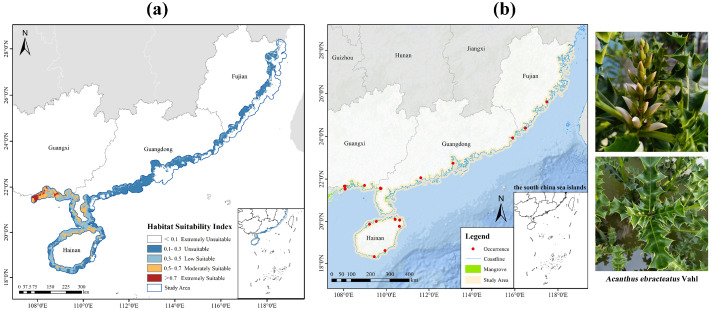
Current potential suitable habitat and occurrence records of *Acanthus ebracteatus*. (**a**) Predicted distribution of potentially suitable habitats. (**b**) Species occurrence records used for model training.

The geographical distribution of species occurrence records used for model training is shown in Figure 2b. These records show strong agreement with the predicted suitability map (Figure 2a), with most presence points falling within the predicted suitable areas. The current potential suitable habitat of *A. ebracteatus* totals 29,320 km^2^, accounting for 29.79% of the study area. It should be noted that all geographical locations described below refer to relative positions within the defined study area. Highly suitable areas comprise 1446 km^2^ (1.47% of the study area) and are concentrated in western and central Guangxi. Moderately suitable areas account for 9274 km^2^ (9.42% of the study area), occurring mainly in western and central Guangxi, with smaller portions located in the western Leizhou Peninsula and Zhanjiang Bay region of Guangdong, as well as in northern Hainan. Low-suitability areas cover 18,600 km^2^ (18.90% of the study area) and are predominantly found in central and eastern Guangxi; across most of the Leizhou Peninsula; and in northern, western, and southern Hainan, as well as in small parts of Fujian.

### 2.4. Spatiotemporal Dynamics of Future Habitats

#### 2.4.1. Distribution Patterns of Potential Suitable Habitats Under Future Climate Scenarios

**Figure 3:**
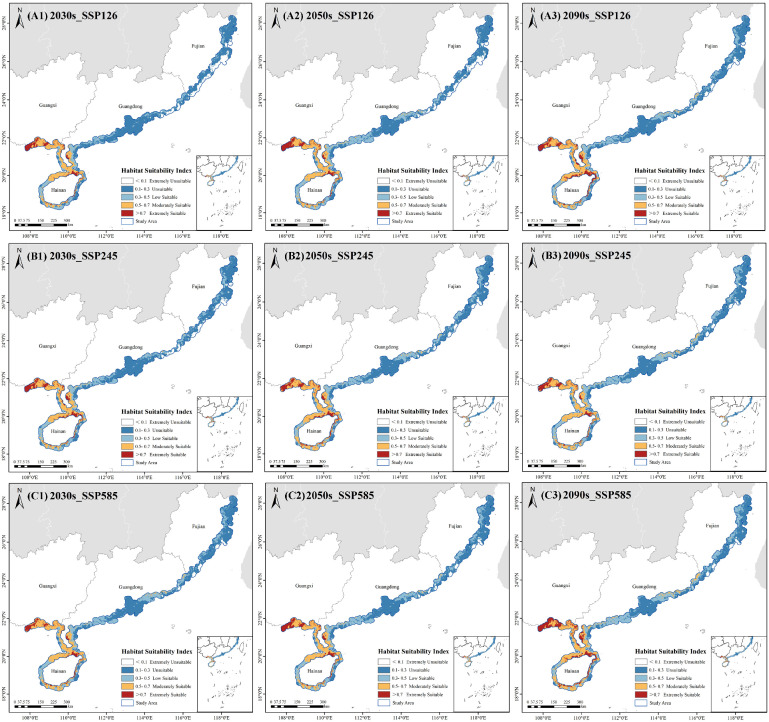
Predicted potential suitable habitats for *Acanthus ebracteatus* under different SSP scenarios (SSP126, SSP245, and SSP585) for the 2030s, 2050s, and 2090s.

As a species endemic to tropical and subtropical intertidal zones, *A. ebracteatus* is highly sensitive to climate change. Using the MaxEnt model, we simulated the distribution of its potential suitable habitats under three shared socioeconomic pathways (SSP126, SSP245, and SSP585) for the 2030s, 2050s, and 2090s (Figure 3). The results revealed a consistent expansion on trend and a significant increase in total suitable areas under all three SSP scenarios over the long term.

Compared to the current baseline period (1970–2000), the most prominent expansion occurred in the 2030s. The potentially suitable areas under SSP126, SSP245, and SSP585 accounted for 42.52%, 48.00%, and 52.62% of the total area of the study area, respectively. The overall expansion patterns varied among scenarios, whereby the SSP126 scenario showed a steady increase in suitable area; the SSP245 scenario exhibited the largest expansion of high- and medium-suitability areas in the 2050s, with highly suitable areas reaching 6240 km^2^. However, this abruptly decreased by 20.5% to 5207 km^2^ in the 2090s, suggesting potential climatic anomalies. Meanwhile, the SSP585 scenario demonstrated the most significant expansion, particularly in the 2090s when highly suitable areas reached 9311 km^2^, accounting for 9.46% of the study area.

In conclusion, while SSP585 showed the greatest potential for expansion, the notable fluctuation under SSP245 requires further investigation, while SSP126 presented a more stable trend relating to suitable area increase.

#### 2.4.2. Spatial–Temporal Dynamics of Potential Suitable Areas

**Figure 4:**
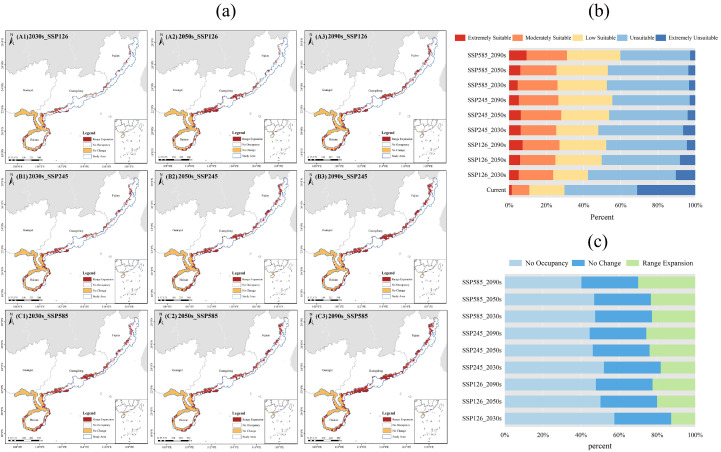
Dynamic changes in potential suitable areas for *Acanthus ebracteatus* under future SSP scenarios. (**a**) Geographical shift in suitable habitats. (**b**) Area changes for each suitability class. (**c**) Net change in suitable area relative to the baseline period.

An analysis of the spatial–temporal dynamics of the potential suitable area for *A. ebracteatus* revealed consistent expansion under all three SSP scenarios, with the magnitude of expansion correlating with increasing CO_2_ concentrations (Figure 4). Relative to the current baseline period (1970–2000), the total suitable area under the SSP126 scenario increased by 12.73%, 20.04%, and 22.47% in the 2030s, 2050s, and 2090s, respectively, demonstrating a stable growth trend. Under the SSP245 scenario, the expansion rates were 18.21%, 24.07%, and 25.77% across the same periods, with the most significant increase occurring in the 2050s. The SSP585 scenario exhibited the greatest expansion, with increases of 22.83%, 23.39%, and 30.07%, culminating in a maximum total suitable area in the 2090s, increasing by 29,550 km^2^ relative to the baseline period. The high-suitability area alone expanded by 7860 km^2^ (Figure 4c).

Geographically, expansion was predominantly concentrated in Maoming, Yangjiang, Huizhou, and Shanwei in Guangdong Province, as well as Lingshui and Changjiang in Hainan Province. Over the long term, low-suitability areas are projected to expand northward to Fujian Province, potentially forming new suitability areas in Shantou and Chaozhou in Guangdong Province, as well as Zhangzhou, Xiamen, and Quanzhou in Fujian Province (Figure 4a). Continuous monitoring is essential to confirm the potential presence of *A. ebracteatus* in these regions. These patterns underscore the complex ecological responses of mangrove species under high-emission scenarios, as well as emphasizing the significant impact of climate warming on the distribution and expansion of *A. ebracteatus*, providing critical references for future conservation and management efforts.

#### 2.4.3. Shifts in the Geographic Centroid of Suitable Habitats

**Figure 5:**
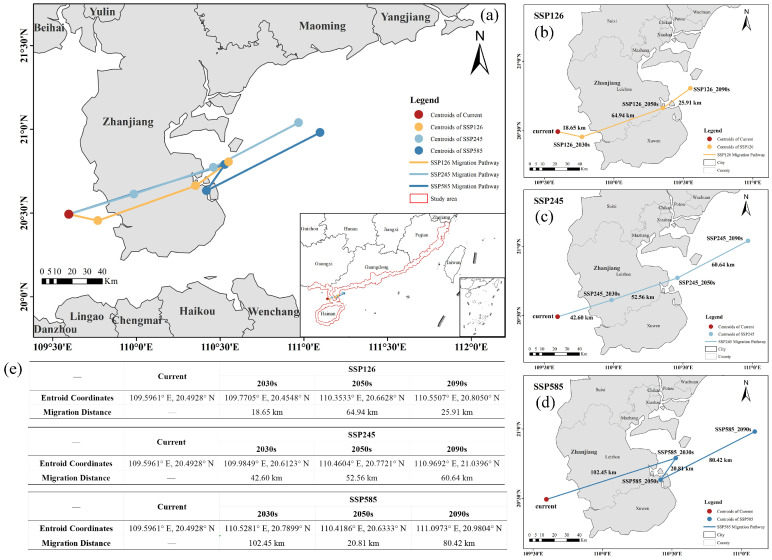
Migration trajectories of the distribution centroid for *Acanthus ebracteatus* under future SSP scenarios. (**a**) Migration trajectories under all three scenarios. (**b**) SSP126 trajectory. (**c**) SSP245 trajectory. (**d**) SSP585 trajectory. (**e**) Summary of centroid coordinates and cumulative migration distances.

The geographic centroid represents the weighted center of modeled habitat suitability, indicating the spatial concentration of potential distribution rather than actual population centers. An analysis of centroid migration patterns for *A. ebracteatus* revealed consistent northeastward shifts under all three SSP scenarios (Figure 5a), reflecting climate-driven habitat expansion toward higher latitudes in response to regional warming. The baseline distribution centroid is located on the western Leizhou Peninsula (109.5961° E, 20.4928° N).

Under the SSP126 scenario (Figure 5b), the centroid is projected to shift slightly southeastward by the 2030s before continuous northeastward through the 2090s, with the longest migration distance (approximately 64.94 km) occurring between the 2030s and 2050s; the trajectory passes Xuwen Beili Island in Zhanjiang City and ultimately reaches the vicinity of Zhanjiang Bay (110.9692° E, 21.0396° N). Under the SSP245 scenario (Figure 5c), a continuous northeastward migration is projected from the 2030s to the 2090s, culminating in a total displacement of approximately 155.80 km past Leizhou City in Zhanjiang City to the eastern waters of the Leizhou Peninsula (110.9692° E, 21.0396° N). Under the SSP585 scenario (Figure 5d), the centroid is expected to migrate the farthest northeastward (approximately 102.45 km) by the 2030s, approaching Zhanjiang Bay, then shift temporarily northwestward toward Xunwen Xinliao Island in the 2050s before moving northeast again in the 2090s to the eastern waters of the Leizhou Peninsula (111.0973° E, 20.9804° N), reaching an endpoint similar to that under the SSP245 scenario by the 2090s.

### 2.5. Novelty and Uncertainty in Future Climate Projections

**Figure 6:**
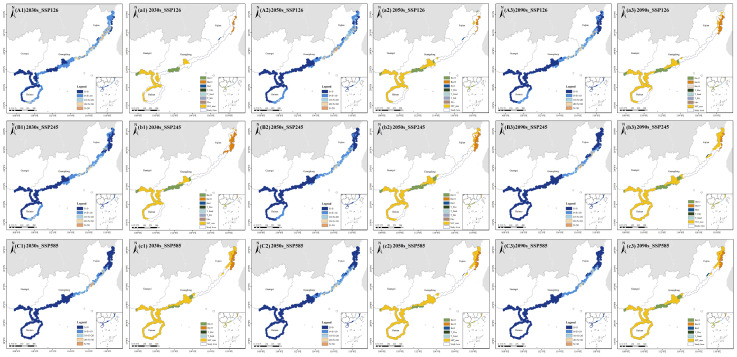
Analysis of MESS and MoD for *Acanthus ebracteatus* under future climate scenarios. (**A1**–**A3**) MESS for SSP126 scenario. (**a1**–**a3**) MoD for SSP126 scenario. (**B1**–**B3**) MESS for SSP245 scenario. (**b1**–**b3**) MoD for SSP245 scenario. (**C1**–**C3**) MESS for SSP585 scenario. (**c1**–**c3**) MoD for SSP585 scenario.

Climatic anomalies (S < 0) within the potentially suitable habitat of *A. ebracteatus* increased over time under all three SSP scenarios (Figure 6). The SSP126 scenario exhibited the smallest deviation from current conditions, with a mean multivariate environmental similarity surface (MESS) value of −5.25, which is significantly higher than those of SSP245 (−14.52) and SSP585 (−19.65). The SSP585 scenario showed the most severe climatic novelty, with over 77% of the study area experiencing novel conditions (S < 0) across all time periods, indicating the strongest departure from current climate baselines. Spatially, novel climates were predominantly projected at the southwest and northern margins of the current suitable habitat. These areas of high climatic novelty align with the projected regions of habitat expansion identified in our distribution models (Section 2.4), suggesting that the observed range shifts are driven by the emergence of novel climate conditions beyond the species’ current ecological niche. In contrast, regions in the central–northern part of the study area, particularly north of the Pearl River Estuary in Guangdong and south of Xinghua Bay in Fujian, retained a higher climate similarity compared to that at present (Figure 6(A1–A3,B1–B3,C1–C3)), indicating a greater reliability of projections in these core habitats, as well as their potential role as climate refugia.

The most dissimilar variables (MoD) driving these anomalies were consistently identified as maximum sea surface temperature (SST_max), annual precipitation (Bio12), Precipitation of Coldest Quarter (Bio19), and Mean Diurnal Range (Bio2) across all three SSP scenarios (Figure 6(a1–a3, b1–b3, c1–c3)). This identification of thermal and moisture variables as the primary drivers of climatic novelty provides a mechanistic explanation for the projected habitat changes reported in Section 2.4. The consistent prominence of these variables across emission scenarios underscores the critical role of temperature and precipitation changes in shaping the future distribution of *A. ebracteatus*.

### 2.6. Conservation Priorities and Gaps Analysis

**Figure 7:**
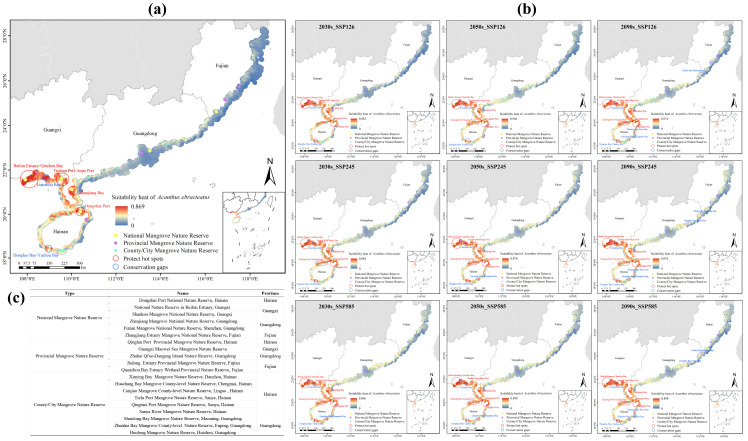
Identification of conservation hotspots and gaps for *Acanthus ebracteatus*. (**a**) Current hotspots and gaps. (**b**) Projected hotspots and gaps under future climate scenarios. (**c**) Mangrove nature reserves.

The spatial distribution of existing mangrove protected areas within the study area was first analyzed, revealing a network of six national, five provincial, and nine municipal and county-level mangrove nature reserves (Figure 7c). We employed Kernel Density Estimation (KDE) to map current habitat suitability hotspots for *A. ebracteatus* (Figure 7a), which identified key areas including Beilun Estuary–Qinzhou Bay, Tieshan Port–Anpu Port, Zhanjiang Bay, and Dongzhai Port. Overlay analysis revealed that although these major hotspots are generally well-covered by the existing protection network, significant gaps persist in highly suitable yet unprotected areas, primarily in Lianzhou Bay and Dongluo Bay–Yazhou Bay.

Future projections under the three SSP scenarios were similarly analyzed using KDE to identify emerging patterns. Both SSP245 and SSP585 scenarios showed expanded hotspot areas compared to current distributions. Newly identified hotspots included Xingying Port–Xiuying Port and Qinglan Port, which were adjacent to the priority hotspot areas. Concurrently, new conservation gaps were projected to emerge, mainly located in Qingshui Bay–Xiangshui Bay, Shanqin Bay–Boao Bay, Honghai Bay–Jieshi Bay, and Zhelin Bay–Dacheng Bay (Figure 7b). These areas, characterized by persistently high habitat suitability across future climate scenarios but inadequate coverage in the current protection network, represent critical conservation priorities.

## 3. Discussion

### 3.1. Effects of Key Environmental Factors on the Distribution of A. ebracteatus

Our integrated MaxEnt and OPGD modeling framework identifies salinity, temperature, and soil composition as the primary determinants of the potential distribution of *A. ebracteatus*. Among these, salinity emerges as a critical limiting factor, which is consistent with its established role in mangrove forest distribution [25]. The model revealed a high sensitivity to SSS_range, indicating that stable, low-salinity environments are optimal for the species (Supplementary Figure S2c). This finding is strongly corroborated by physiological evidence. Tong et al. (2024) showed that the seedlings exhibit optimal growth in salinities between 0 and 15, with significant growth inhibition and mortality occurring at salinities exceeding 20 [26]. Hu et al. (2020) also found that *A. ebracteatus* performed well in the low-salinity, high-tide area, while struggling to survive in high-salinity, low-tide areas [27], validating our model’s prediction that environments with high salinity fluctuations are fundamentally unsuitable.

Climatic variables, particularly temperature and precipitation, are also fundamental in shaping the species’ habitat suitability [19]. Our analysis indicates that sea surface temperature (SST) exerts a more prominent influence than terrestrial air temperature, a finding that is consistent with regional mangrove distribution models in similar coastal ecosystems [28]. Specifically, the habitat suitability probability for *A. ebracteatus* surpasses 0.5 when the maximum SST_max exceeds 30.9 °C (Supplementary Figure S2d), confirming the species’ thermophilic nature and explaining its concentration in low-latitude regions. Precipitation (Bio12) exhibited a positive relationship with habitat suitability (Supplementary Figure S2g), likely through its role in diluting seawater salinity [2]. Higher precipitation not only alleviates salinity stress for low-salinity-adapted species, but also promotes seedling survival and establishment, thereby enhancing distribution potential.

Soil serves as the primary carrier of nutrients and water for mangroves and was crucial for the growth of *A. ebracteatus* [29]. The model demonstrate a strong preference for silt-rich substrates (T_silt) and an intolerance to high sand content (T_sand) (Supplementary Figure S2e,f). This edaphic specialization aligns with field observations from the Leizhou Peninsula, where *A. ebracteatus* predominantly colonizes nutrient-rich, deep-mud alluvial flats [11]. The underlying mechanisms relate to fundamental soil properties. Silt provides water retention and nutrient adsorption, which helps mitigate salinity stress. Conversely, sandy substrates are characterized by poor water retention, rapid nutrient leaching, and physical instability, all of which impede root establishment and growth. These findings are supported by the existing literature. Li et al. (2024) identified soil conditions as primary drivers of *A. ebracteatus* distribution [30], while Blanco-Sacristán et al. (2022) demonstrated that four soil factors dominate mangrove distribution in the Red Sea [20]. Collectively, these studies confirm the critical role of soil factors in determining mangrove habitat suitability.

Crucially, the influence of these environmental factors is amplified through their interactions. The OPGD analysis revealed a strong synergistic effect between salinity and soil texture, with the correlation coefficient between SSS_range and both T_Sand and T_Silt reaching 0.8 (Figure 1b). This indicates that the combination of a low salinity and high silt content creates an optimally conducive environment for *A. ebracteatus*. Conversely, the co-occurrence of a high salinity and high sand content compounds environmental stress, severely constraining the species’ distribution.

In summary, the distribution of *A. ebracteatus* is governed by a complex interplay of salinity, temperature, and soil factors. Its ecological adaptation is characterized by low salt tolerance, thermophilic preference, and dependency on nutrient-rich substrates. These ecological requirements are reflected in negative responses to SSS_range and T_Sand, as well as positive responses to SST_max, Bio12, and T_Silt. The strong interactions among these variables further refine habitat suitability. These findings provide scientific evidence for understanding the species’ ecological adaptability and offer theoretical support for targeted conservation strategies.

### 3.2. Response of A. ebracteatus Distribution to Future Climate Change

Our model projections indicate a significant expansion of the potential suitable habitat for *A. ebracteatus* under all three SSP scenarios (Figure 4). This trend is consistent with projections for other mangrove species [31]. This shift can be largely attributed to ongoing climate change. According to the IPCC Sixth Assessment Report (2023) [32], global surface temperatures have already risen by 1.1 °C and are expected to continue rising, particularly under the SSP585 scenario, which may see end-of-century temperature increases of 3.3 to 5.7 °C. Additionally, the intensity and frequency of global extreme precipitation events may increase by 5% to 20%. In northern hemisphere coastal regions, climate change is characterized by a strengthening of the summer monsoon and the weakening of the winter monsoon, leading to increased rainfall [33]. This is corroborated by MESS and MoD analysis (Figure 6), which identified maximum sea surface temperature (SST_max) and precipitation-related variables (Bio12, Bio19) as the primary drivers of future climatic shifts in the study area.

The synergistic effects of rising temperatures and increased precipitation are the key drivers enhancing habitat suitability for *A. ebracteatus* and facilitating its range expansion. Newly identified high-suitability areas are concentrated in the Beilun Estuary, Fangchenggang, Anpu Port, Zhanjiang Bay, and Dongzhai Port (Figure 3). The recent discovery of new *A. ebracteatus* populations in Fangchenggang [10] and the Leizhou Peninsula [11] around 2020 confirmed the ongoing process of habitat expansion in these areas. Furthermore, the consistent northeastward migration of the geographic centroid of the species’ potential distribution is projected across all three scenarios. This poleward migration aligns with the widely documented trend of subtropical mangroves shifting toward higher latitudes in response to global warming [34].

In summary, climate change is a key factor influencing the observed and projected range expansion and poleward migration of *A. ebracteatus*. This study offers a framework for understanding the species’ potential ecological responses to future climate scenarios. To support long-term conservation efforts, it would be valuable to maintain ongoing monitoring processes and to further investigate the species’ capacity to adapt to these changing environmental conditions.

### 3.3. Conservation and Management Recommendations for A. ebracteatus

Currently, the distribution area of *A. ebracteatus* in China is less than 10 hectares, facing a severe risk of regional extinction [35]. The populations show poor growth status and suffer from severe habitat fragmentation [10]. Thus, artificial interventions are urgently needed to safeguard its minimum viable populations. Both in situ and ex situ conservation are essential strategies for its effective protection and recovery [36]. In situ conservation can be guided by hotspot identification and gap analysis, relying primarily on natural regeneration to maintain and restore populations within their native habitats [37]. In the core distribution areas, such as Beilun Estuary–Qinzhou Bay, Tieshan Port–Anpu Port, Zhanjiang Bay, and Dongzhai Port, the KDE results indicate high habitat suitability with the remaining populations present (Figure 7). Therefore, conservation efforts in these regions should focus on habitat protection and ecological restoration, with natural regeneration as the principal approach.

In contrast, regions such as Lianzhou Bay and Dongluo Bay–Yazhou Bay, which exhibit high habitat potential but are not yet included in protected areas, require a combination of ex situ conservation and the establishment of small population reserves. In these areas, artificial introduction and propagation, coupled with long-term management, are necessary to compensate for the limited capacity of natural regeneration and to prevent the loss of rare genetic resources. For future hotspot regions predicted under climate warming, such as Qinglan Port, Xinying Port–Xiuying Port, Shanqin Bay–Boao Bay, Qingshui Bay–Xiangshui Bay, Honghai Bay–Jieshi Bay, and Zhelin Bay–Dachen Bay, where no natural populations currently exist, large-scale reforestation and carefully designed introduction trials should be prioritized. These measures will not only expand the potential distribution of *A. ebracteatus* but also enhance mangrove biodiversity and accelerate the recovery of ecosystem functioning. In summary, conservation strategies should be region-specific, i.e., natural regeneration in core areas, a combination of assisted introduction and small population management in potential zones, and active reforestation with experimental introduction in newly suitable future habitats.

### 3.4. Potential Limitations and Future Research Directions

This study identified the key environmental factors influencing the potential distribution of *A. ebracteatus* and analyzed its response mechanisms. However, due to data limitations, our model focused on the fundamental drivers of climate, soil, topography, and marine conditions but did not incorporate anthropogenic factors, such as land use change and aquaculture development, or infrastructure like seawalls. Furthermore, biotic interactions and species-specific dispersal limitations, which are known to shape realized distributions, were not explicitly modeled. Future research could integrate these variables, potentially using dynamic models like Dyna-CLUE to provide a more comprehensive projection of the species’ future realized niche.

Our projections are based on the MaxEnt and the CMIP6 climate dataset. Although widely validated, any single-model approach is subject to specific structural assumptions. The application of an ensemble modeling framework, which integrates outputs from multiple algorithms, could enhance the robustness and reduce the uncertainty of the projections. Moreover, as datasets continue to update, future predictions should be periodically updated with next-generation climate model outputs to refine our understanding of long-term habitat suitability.

Additionally, it is important to distinguish between the potential distribution predicted by our model and the actual area that the species will occupy. This is because the MaxEnt model characterizes the fundamental ecological niche, which is invariably broader than the realized niche. Although the projections under the three SSP scenarios suggest an expansion trend of suitable habitats, coastal land use complexity and practical constraints including suitable forest land types and land use policies limit real-world habitat availability. Furthermore, barriers such as seawalls and sea-level rise may also prevent the species from colonizing otherwise suitable areas [38].

To help align these projections with practical conservation needs, we recommend long-term monitoring and targeted field surveys, especially in newly identified high-suitability areas. Such ground-based efforts would contribute to validating model predictions, improve our understanding of the species’ adaptive capacity, and support the design of more effective mangrove restoration and management strategies under climate change.

## 4. Materials and Methods

### 4.1. Species Occurrence Data and Study Area

*A. ebracteatus* is an endangered mangrove species. We compiled distribution data from the Global Biodiversity Information Facility (GBIF Occurrence Download Available online: https://doi.org/10.15468/dl.yqvmhu, accessed on 25 September 2024), the National Specimen Information Infrastructure (NSII), the Chinese National Plant Specimen Resource Center (CVH), and the published literature. To supplement these data, a field survey was conducted in Anpu Port on the Leizhou Peninsula, combining UAV reconnaissance with quadrat sampling to collect primary occurrence data and validate previously recorded distributions. For records lacking precise coordinates, geographic coordinates were georeferenced using the Baidu Maps coordinate pickup system (http://api.map.baidu.com/, accessed on 12 October 2024), yielding a total of 130 occurrence records. Based on mangrove growth characteristics and expert consultation, spurious or erroneous records were excluded. To mitigate model overfitting and minimize spatial sampling bias, occurrence data were spatially rarefied using the SDMtoolbox [39] in ArcMap 10.8, retaining a single record per 1 km^2^ grid cell. A final set of 26 spatially independent occurrence records was obtained for *A. ebracteatus* (Figure 2b; Supplementary Table S1). These final occurrence data were exported in CSV format to be input into the MaxEnt model.

Mangrove forests in China are primarily distributed between 18° N to 30° N and 105° E to 125° E [40]. Based on the current distribution of *A. ebracteatus*, this study defined the study area to encompass the coastal zones of Hainan, Guangdong, Guangxi, and Fujian provinces, which comprehensively covers the species’ known distribution range in China. Following the “Simplified Procedures for the Comprehensive Survey of Coastal and Tidal Flat Resources in China,” the inland boundary was set at 10 km from the coastline, while the seaward boundary extended to the 10–15 m isobath. Drawing on established definitions of mangrove habitat space [41,42], a total buffer width of 20 km (10 km on both sides of the coastline) was adopted to delineate the study area.

### 4.2. Environment Variables and Processing

Mangrove species inhabit the dynamic ecotone between land and sea, where their distribution is influenced by a complex suite of environmental factors. Although responses vary among species, key determinants typically include temperature, salinity, precipitation, elevation, and soil properties [27,43]. Accordingly, this study initially considered 38 environmental variables from five categories. These included 19 bioclimatic variables and 1 topographic variable at 30 arc-second resolution from WorldClim (version 2.1; www.worldclim.com, accessed on 12 October 2024); 10 soil variables from the Harmonized World Soil Database (version 2.0; http://www.fao.org, accessed on 22 October 2024); and 8 marine variables, comprising 4 sea surface temperature and 4 sea surface salinity layers, at 5 arc-minute resolution from the Bio-ORACLE database (version 3.0; https://bio-oracle.org, accessed on 1 December 2024).

To address the integrated influence of terrestrial and marine drivers on mangrove distribution, multi-source spatial data were harmonized within ArcMap 10.8. Processing included (1) masking all layers to the study area extent and applying Kriging interpolation to ensure continuous spatial coverage where necessary [30]; (2) resampling all marine variables to a consistent 30 arc-second resolution using bilinear interpolation; and (3) converting all environmental layers to ASCII format for compatibility with MaxEnt.

To mitigate multicollinearity and model overfitting, an initial screening was conducted using MaxEnt V3.4.1 [44]. Variables with zero contribution, as identified by Jackknife testing, were excluded. Subsequently, the values of the remaining 18 variables were extracted from species occurrence records using the raster package [45] in R 4.3.3. A Spearman correlation analysis was then performed using the cor() function [46], and results were visualized via the corrplot package [47] (Supplementary Figure S3a). From highly correlated variable pairs (|r| > 0.75), the variable with greater ecological relevance and higher permutation importance was retained. This process resulted in a final set of 10 environmental variables for formal modeling (Table 2, Supplementary Figure S3b).

To develop a scientifically informed conservation strategy for *A. ebracteatus* under climate change, this study projected its spatial distribution across three future time periods—the near-future (2030s; average of 2020–2040), mid-century (2050s; average of 2040–2060), and end-of-century (2090s; average of 2080–2100) periods. Future climate projections were derived from the BCC-CSM2-MR model (CMIP6) developed by the National Climate Center of China [48]. Three Shared Socioeconomic Pathways (SSPs)were used: SSP126, SSP245, and SSP585, representing low, medium, and high radiative forcing scenarios, respectively. Corresponding future sea surface temperature and sea surface salinity data were consistently obtained from these model scenarios to maintain climatic and methodological consistency.

### 4.3. Model Parameter Optimization and Evaluation

To optimize model performance, we adjusted the regularization multiplier (RM) and feature combinations (FCs) using the ENMeval 2.0 package [24] in R 4.4.3. The RM controls the complexity of the model, while FCs describe the extent to which environmental variables influence species distribution [49]. By experimenting with different function combinations, the model can better capture the complex relationships between environmental variables and ecological distribution [18]. RM values ranged from 0.5 to 4.0 (intervals of 0.5), and 6 FCs (L, H, LQ, LQH, LQHP, and LQHPT) were tested, resulting in 48 candidate models. The Akaike information criterion (delta.AICc) [50] was applied to identify the optimal parameter combination that balanced model fit and complexity, with delta.AICc = 0 indicating the optimal combination. Overfitting was assessed according to the difference between training and testing AUC (AUC.diff) [51].

Model evaluation was based on 26 occurrence records and 10 environmental predictors. Using the Bootstrap method, 75% of the records were randomly assigned to the training dataset and 25% to the testing dataset, with 10 replicates being conducted in logistic output format. Predictive accuracy was assessed with the receiver operating characteristic (ROC) curve and the area under the curve (AUC), where higher AUC values indicated greater predictive power [52]. The evaluation criteria are as follows: poor (0.6–0.7), fair (0.7–0.8), good (0.8–0.9), and excellent (0.9–1) [18].

To evaluate the relative importance of environmental variables, we employed three complementary approaches: Jackknife testing, percentage contribution, and permutation importance. Response curves were generated to examine the ecological thresholds of key variables. In the Jackknife test, variables whose exclusion led to substantial decreases in AUC or training gain were considered most influential in determining habitat suitability.

### 4.4. Identifying Key Environmental Drivers and Interactions

The geographic detector model is an effective method for analyzing spatial hierarchical heterogeneity, while the optimal parameter geographic detector (OPGD) determines the optimal parameter combination by optimizing spatial data discretization and spatial analysis scales to enhance analytical accuracy [22]. In this study, the “GD” package [22] was run using R 4.4.3 to conduct OPGD analysis, with environmental factors as independent variables and the suitability index for suitable habitats as the dependent variable. Both factor detectors and interaction detectors were employed. The factor detector assesses the relative importance of variables using the *Q* statistic, with the q-value reflecting the extent of their influence on the suitable habitats of *A. ebracteatus*, thereby identifying dominant factors. The interaction detector compares the q-values of single and dual factors in order to analyze the type of interaction between the two factors, including the following five types: nonlinear weakening, single-factor weakening, bifactor enhance, independent, and nonlinear enhance [53]. This method effectively reveals the impact of environmental factor interactions on the spatial differentiation of suitable habitats for *A. ebracteatus*.

### 4.5. Spatiotemporal Dynamics of Habitat Suitability

#### 4.5.1. Habitat Suitability Mapping and Classification

The continuous habitat suitability maps generated by MaxEnt (in ASCII format) were processed using ArcMap 10.8. For visualization and spatial contextualization, the raw outputs were first converted to the TIFF format and overlaid on a map of China’s administrative divisions. To facilitate further quantitative analysis, these maps were then reclassified into four distinct suitability levels based on their probability values. This classification was determined by integrating the natural breaks (Jenks) method, with considerations of the species’ distribution characteristics and reference to previous classifications of mangrove habitat suitability zones [21]. The habitats were thus categorized as follows: High Suitability (*p* ≥ 0.7), Moderate Suitability (0.5 ≤ *p* < 0.7), Low Suitability (0.3 ≤ *p* < 0.5), and Unsuitable (*p* < 0.3). The total area for each category was subsequently quantified.

#### 4.5.2. Quantification of Habitat Dynamics: Changes in Area and Centroid Shift

The dynamics of the potential habitat were quantified by analyzing both the total area change and the shift in the core distribution centroid. For assessing overall expansion or contraction, the total suitable area was defined as all regions with a suitability probability (*p*) ≥ 0.3 (i.e., combining the Low, Moderate, and High classes defined in 4.5.1). Using the SDMtoolbox [39] in ArcMap 10.8, we created a binary map (suitable = 1, unsuitable = 0) from which the total area was calculated for the current period and all future scenarios to determine the net changes.

Furthermore, to track the movement of the most viable population centers, the analysis focused on the core habitat, defined as areas with Moderate and High suitability (*p* ≥ 0.5). The geographic centroid of this core habitat was calculated for each period using the ‘Measuring Geographic Distributions’ tool in ArcMap 10.8. The migration distance and direction of the centroid were then analyzed to reveal trends in the species’ potential distribution shift.

#### 4.5.3. Assessment of Climate Novelty in Future Projections

To evaluate the uncertainty and reliability of our future projections, we conducted Multivariate Environmental Similarity Surface (MESS) and Most Dissimilar Variable (MoD) analyses. These analyses quantify where and to what extent future climate conditions deviate from the current baseline, thereby identifying regions where model projections are extrapolative and less reliable.

The MESS analysis calculates a similarity index (S) for each future grid cell relative to the current climate reference layer. An S value > 0 indicates analogous conditions, with higher values denoting greater similarity. An S value < 0 indicates a novel climate, where at least one variable exceeds the current range of values; more-negative values signify greater divergence. For novel climates (S < 0), the MoD analysis identifies the specific environmental variable that contributes most to this dissimilarity, thus highlighting the key climatic drivers of distribution change.

All MESS and MoD analyses were performed in MaxEnt 3.4.3 using the ‘Density.tools.Novel’ tool [54], with the current climate conditions set as the reference layer. This process allowed us to map the spatial patterns of climate novelty under each future scenario and period, as well as allowing us to identify the primary variables responsible for these changes.

### 4.6. Analysis of Conservation Priorities and Gaps

To assess the conservation effectiveness of *A. ebracteatus*, we first generated a potential distribution hotspot map using Kernel Density Estimation (KDE) in ArcMap 10.8, based on the habitat suitability results. To weight the analysis according to habitat quality, the continuous habitat suitability map for the current period was used as the weighting factor in the KDE calculation. Regions with the highest hotspot values were delineated as priority conservation areas.

We then evaluated the species’ conservation status by spatially overlaying these priority areas with the current distribution of mangrove nature reserves. This overlay analysis enabled the identification of conservation gaps, which are high-priority habitats that fall outside the existing protected area network [28]. This assessment provides a critical foundation for developing targeted conservation strategies for *A. ebracteatus*.

## 5. Conclusions

In this study, we integrated MaxEnt and OPGD models to simulate the potential suitable habitat distribution of an endangered mangrove species *A. ebracteatus* under current and future climate scenarios (SSP126, SSP245, and SSP585), as well as systematically analyzing the influence of key environmental factors on its spatial pattern. The results indicate that the distribution of this species is primarily driven by sea surface salinity range (SSS_range), maximum sea surface temperature (SST_max), soil texture (T_Silt and T_Sand), and annual precipitation (Bio12). Among these, SSS_range was identified as the core limiting factor. The OPGD interaction detection analysis further revealed a significant synergistic enhancement between SSS_range and other environmental variables, particularly soil properties (q > 0.8). These findings not only deepen the understanding of the species’ ecological niche but also emphasize that low-salinity, suitable temperature, and silt-rich site conditions must be considered as essential criteria for in situ and ex situ conservation planning.

Under the three SSP scenarios, driven by global warming, the potential suitable habitat of *A. ebracteatus* is projected to expand continuously, with the distribution centroid shifting northeastward from the current location (109.5961° E, 20.4928° N) to a maximum of 110.9692° E, 21.0396° N. This poleward migration is mainly attributed to increasing temperature and altered precipitation patterns, which is consistent with the general response of mangrove ecosystems to climate change.

From a conservation perspective, existing protected areas do not fully cover all current and future high-suitability regions. Based on KDE analysis, we identified key priority conservation areas such as Beilun Estuary–Qinzhou Bay, Tieshan Port–Anpu Port, and Dongzhai Port–Qinglan Port, as well as critical conservation gaps including Lianzhou Bay and Dongluo Bay–Yazhou Bay. Consequently, we recommend a hierarchical and region-specific conservation strategy. For existing core distribution areas, efforts should focus on strengthening in situ protection and promoting natural regeneration to ensure the stability of core populations. For regions projected to have high potential but currently lacking protection, the implementation of small-population conservation and restoration measures is advisable. Furthermore, in new areas that are currently unoccupied but predicted to become highly suitable, mangrove restoration and experimental introduction programs should be prioritized to facilitate the species’ natural range expansion.

In summary, this study provides a foundational understanding of the ecological drivers of *A. ebracteatus* and their response to future climate. Subsequent research should integrate multi-model integration methods, incorporate additional variables, and include field validation to further enhance the reliability of these predictions and to guide more refined conservation practices.

## Figures and Tables

**Table 1:** The contribution proportions and cumulative proportion contributions of environmental variables.

Variable	Percent Contribution (%)	Permutation Importance (%)
SSS_range	36.4	40.0
SST_max	19.0	11.7
T_Silt	17.2	17.6
T_Sand	10.0	3.8
Bio12	8.3	7.3
Bio14	3.4	6.2
T_Elec	2.5	3.0
Bio2	1.5	3.5
Bio19	1.0	5.2
Elev	0.6	1.7

**Table 2:** Factors used in model prediction.

Type	Code	Factor Description	Unit
Bioclimate	Bio2	Mean Diurnal Range	°C
	Bio12	Annual Precipitation	mm
	Bio14	Precipitation of Driest Month	mm
	Bio19	Precipitation of Coldest Quarter	mm
Topographic	Elev	Elevation	m
Soil	T_Sand	Topsoil Sand	%
	T_Silt	Topsoil Silt	%
	T_Elec	Topsoil Electric Conductivity	dS/m
Sea Surface Temperature	SST_max	Max Sea Surface Temperature	°C
Sea Surface Salinity	SSS_range	Range of Sea Surface Salinity	PSU

## Data Availability

Data are contained within the article and Supplementary Materials.

## Supplementary Materials

The following supporting information can be downloaded at: https://www.mdpi.com/article/10.3390/plants14182827/s1, Figure S1: ENMeval-based parameter optimization for the MaxEnt model, (a) Delta.AICc across parameter settings, (b) AUC.diff across parameter settings; Figure S2. Results of the Jackknife test and response curves of key environmental variables from the MaxEnt model for *Acanthus ebracteatus*, (a,b) Jackknife test, (c–g) Response curves; Figure S3: Correlation analysis of environmental factors, (a) Heat map showing the correlation between 18 environmental factors, (b) Matrix diagram showing the correlation between 10 environmental factors; Table S1: Georeferenced occurrence records of Acanthus ebracteatus after spatial filtering for Maxent modelling.
