# Supplementary material for: Predicting Potential Habitats of the Endangered Mangrove Species Acanthus ebracteatus Under Current and Future Climatic Scenarios Based on MaxEnt and OPGD Models
Source: Plants (Basel). 2025 Sep 10;14(18):2827. doi: 10.3390/plants14182827 (PMC12473658; doi:10.3390/plants14182827)
Supplement: Supplementary file 1 [file plants-14-02827-s001.zip › plants-3771725-supplementary.pdf]

Supplementary Materials

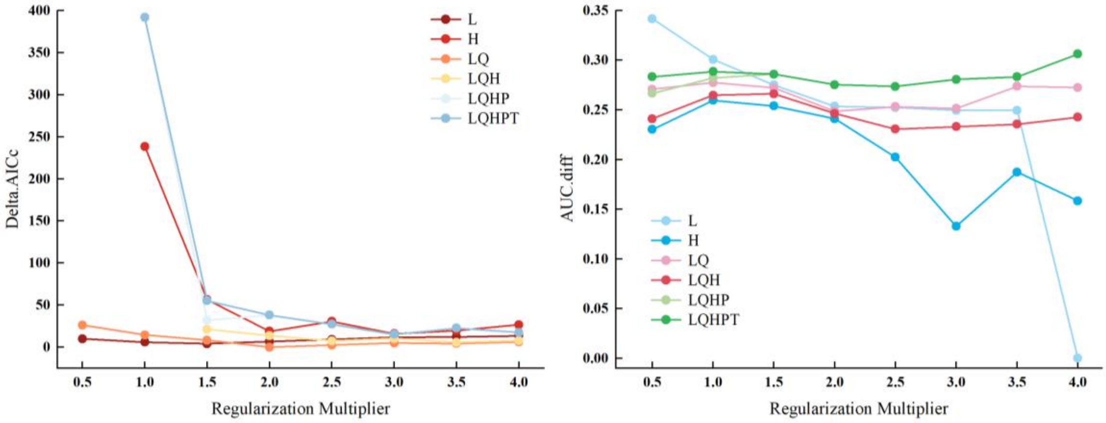

Supplementary Figure S1. ENMeval-based parameter optimization for the MaxEnt model, (a) Delta.AICc across parameter settings, (b) AUC.diff across parameter settings.

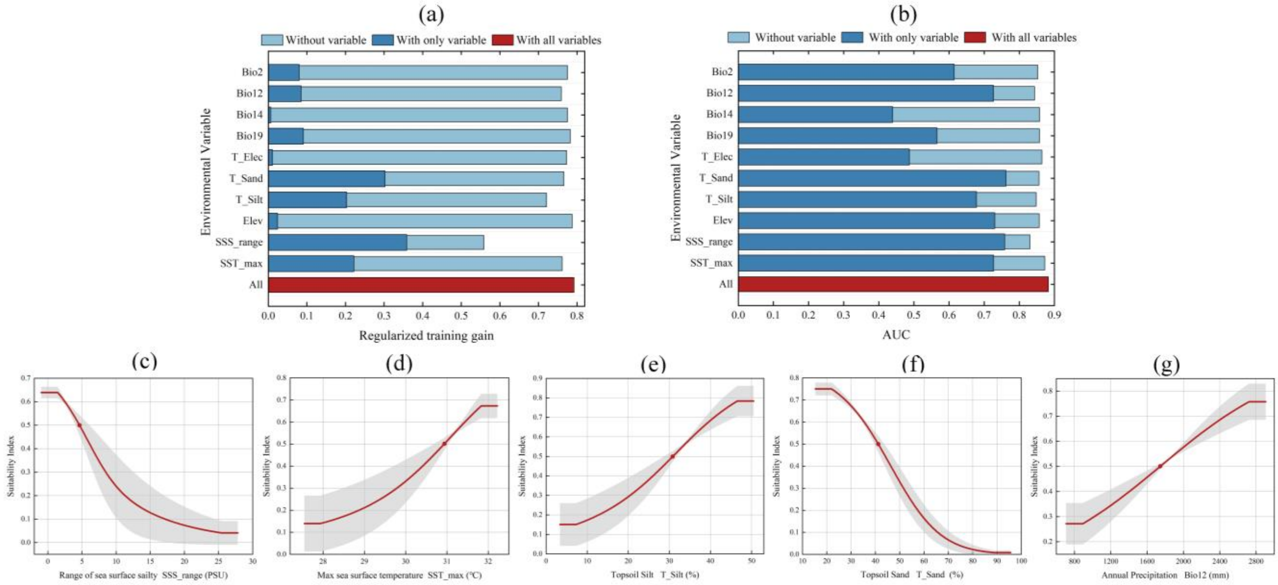

Supplementary Figure S2. Results of the Jackknife test and response curves of key environmental variables from the MaxEnt model for *Acanthus ebracteatus*, (a,b) Jackknife test, (c-g) Response curves.

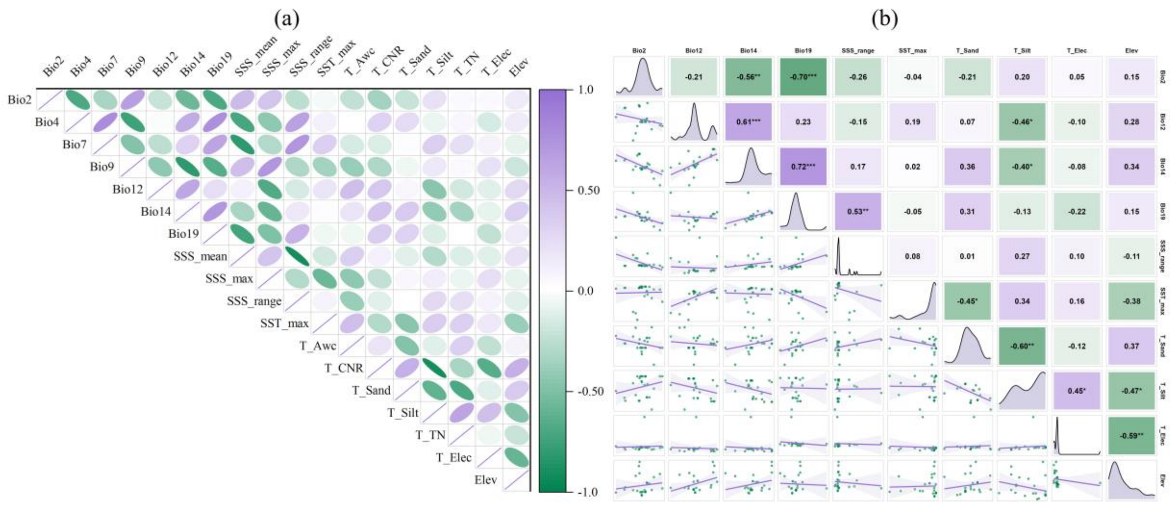

Supplementary Figure S3. Correlation analysis of environmental factors, (a) Heat map showing the correlation between 18 environmental factors, (b) Matrix diagram showing the correlation between 10 environmental factors.

Supplementary Table S1.Georeferenced occurrence records of Acanthus ebracteatus after spatial filtering for Maxent modelling.

Number	Location	Source
1	117.4300, 23.7000	GBIF
2	109.6800, 19.9200	GBIF
3	108.2200, 21.5000	GBIF
4	113.6348, 22.4224	GBIF
5	116.7300, 23.3200	GBIF
6	110.6000, 19.9700	GBIF
7	118.6800, 24.7800	GBIF
8	110.8200, 19.9200	GBIF
9	109.5041, 18.2520	GBIF
10	110.0290, 18.5167	GBIF
11	111.9818, 21.8613	GBIF
12	109.1882, 21.6463	NSII
13	109.3504, 19.8023	NSII
14	110.8184, 19.9284	CVH
15	110.6014, 19.9852	CVH
16	108.2238, 21.6506	CVH
17	108.2261, 21.5163	Published literature
18	108.1931, 21.6462	Published literature
19	108.2274,21.6500	Published literature
20	110.7871, 19.6278	Published literature
21	109.9886, 21.4634	Published literature
22	109.9736, 21.4621	Published literature
23	109.9858, 21.4652	Published literature
24	109.9919, 21.4634	Published literature

Number	Location	Source
25	109.9598, 21.4848	Field survey
26	109.9705, 21.4689	Field survey
